# Spatial epitranscriptomics reveals A-to-I editome specific to cancer stem cell microniches

**DOI:** 10.1038/s41467-022-30299-3

**Published:** 2022-05-09

**Authors:** Amos C. Lee, Yongju Lee, Ahyoun Choi, Han-Byoel Lee, Kyoungseob Shin, Hyunho Lee, Ji Young Kim, Han Suk Ryu, Hoe Suk Kim, Seung Yeon Ryu, Sangeun Lee, Jong-Ho Cheun, Duck Kyun Yoo, Sumin Lee, Hansol Choi, Taehoon Ryu, Huiran Yeom, Namphil Kim, Jinsung Noh, Yonghee Lee, Inyoung Kim, Sangwook Bae, Jinhyun Kim, Wooseok Lee, Okju Kim, Yushin Jung, Changhoe Kim, Seo Woo Song, Yeongjae Choi, Junho Chung, Byung Gee Kim, Wonshik Han, Sunghoon Kwon

**Affiliations:** 1 Bio-MAX Institute, Seoul National University, Seoul, 08826 Republic of Korea; 2 Department of Electrical and Computer Engineering, Seoul National University, Seoul, 08826 Republic of Korea; 3 Interdisciplinary Program in Bioengineering, Seoul National University, Seoul, 08826 Republic of Korea; 4 Department of Surgery, Seoul National University College of Medicine, Seoul, 03080 Republic of Korea; 5 Biomedical Research Institute, Seoul National University Hospital, Seoul, 03080 Republic of Korea; 6 Cancer Research Institute, Seoul National University, Seoul, 03080 Republic of Korea; 7 Department of Pathology, Seoul National University College of Medicine, Seoul, 03080 Republic of Korea; 8 Interdisciplinary Programs in Cancer Biology Major, Seoul National University Graduate School, Seoul, 03080 Republic of Korea; 9 Integrated Major in Innovative Medical Science, Seoul National University Graduate School, Seoul, 03080 Republic of Korea; 10 Department of Biochemistry and Molecular Biology, Seoul National University College of Medicine, Seoul, 03080 Republic of Korea; 11 Department of Biomedical Science, Seoul National University College of Medicine, Seoul, 03080 Republic of Korea; 12 ATG LIfetech Inc, Seoul, 08507 Republic of Korea; 13 Artificial Intelligence Institute, Seoul National University, Seoul, 08826 Republic of Korea; 14 Celemics, Inc, Seoul, 08506 Republic of Korea; 15 School of Materials Science and Engineering, Gwangju Institute of Science and Technology (GIST), Gwangju, 61105 Republic of Korea; 16 Institute of Molecular Biology and Genetics, Seoul National University, Seoul, 08826 Republic of Korea; 17 School of Chemical and Biological Engineering, Seoul National University, Seoul, 08826 Republic of Korea; 18 BK21+ Creative Research Engineer Development for IT, Seoul National University, Seoul, 08826 Republic of Korea; 19 Institutes of Entrepreneurial BioConvergence, Seoul National University, Seoul, 08826 Republic of Korea; 20 Present Address: Department of Surgery, SMG-SNU Boramae Medical Center, Seoul, 03080 Republic of Korea

**Keywords:** RNA sequencing, Breast cancer, Cancer genomics, RNA editing

## Abstract

Epitranscriptomic features, such as single-base RNA editing, are sources of transcript diversity in cancer, but little is understood in terms of their spatial context in the tumour microenvironment. Here, we introduce spatial-histopathological examination-linked epitranscriptomics converged to transcriptomics with sequencing (Select-seq), which isolates regions of interest from immunofluorescence-stained tissue and obtains transcriptomic and epitranscriptomic data. With Select-seq, we analyse the cancer stem cell-like microniches in relation to the tumour microenvironment of triple-negative breast cancer patients. We identify alternative splice variants, perform complementarity-determining region analysis of infiltrating T cells and B cells, and assess adenosine-to-inosine base editing in tumour tissue sections. Especially, in triple-negative breast cancer microniches, adenosine-to-inosine editome specific to different microniche groups is identified.

## Abstract (web summary)

The spatial context of epitranscriptomic features in the tumour microenvironment remains poorly understood. Here, a method for transcriptomic and epitranscriptomic analysis of immunofluorescence-stained tissue, Select-seq, is applied to stem cell-like microniches in triple negative breast cancer.

## Introduction

The tumour microenvironment (TME) contains microniches with spatially heterogeneous transcriptomic and epitranscriptomic features, such as alternative splicing^1^ or non-synonymous single-base RNA editing^2,3^. One dynamic epitranscriptomic modification that generates transcript diversity in microniches is adenosine deaminases acting on RNA (ADAR) enzyme-mediated adenosine-to-inosine (A-to-I) editing^4^, including changes in translated and untranslated regions that affect the TME functionally and pathologically^5^. Thus, single-base-resolution epitranscriptomic analysis of the tumour microniches is the key to understanding how A-to-I editing affects the tumour. In addition, to characterize the microniches, spatial transcriptomic data must be accompanied by spatial epitranscriptomics data. Several technologies that enable gene expression analysis within histopathological and spatial contexts^6–8^ have been applied to depict the spatial landscape of the tumour^9,10^. However, spatially barcoded transcripts and in situ barcodes in spatial transcriptomic methodologies use only fractions of the full-length transcriptome due to limitations in reading length in most widely adopted low error next-generation sequencing (NGS), which makes it difficult to analyze epitranscriptomic information, such as alternative splicing or A-to-I editome. Recent studies incorporated microarray-based spatial technology for long-read NGS to realize the full-length spatial transcriptome after spatial barcoding^11,12^, but the sequencing accuracy of long-read NGS for distinguishing single-base variants is not yet comparable to that of short-read NGS^13^. Thus, investigation of the epitranscriptomic features in each intratumoural microniche necessitates simultaneous multi-modal analysis of transcriptomic, epitranscriptomic and spatial information at single-base resolution. Moreover, there were methodological difficulties that laser capture microdissection (LCM) that can isolate ROIs from IF-stained tissues^14,15^ need to overcome for spatial epitranscriptome analysis. Spatial epitranscriptome (as well as immune cell receptor sequences) require a sizable number of samples and high-quality transcriptome data to secure the statistical importance of rare and single-nucleotide level event. Also, RNA is a very fragile material that continues to degrade and the quality can drop by 70% within an hour^16^.

**Fig. 1:**
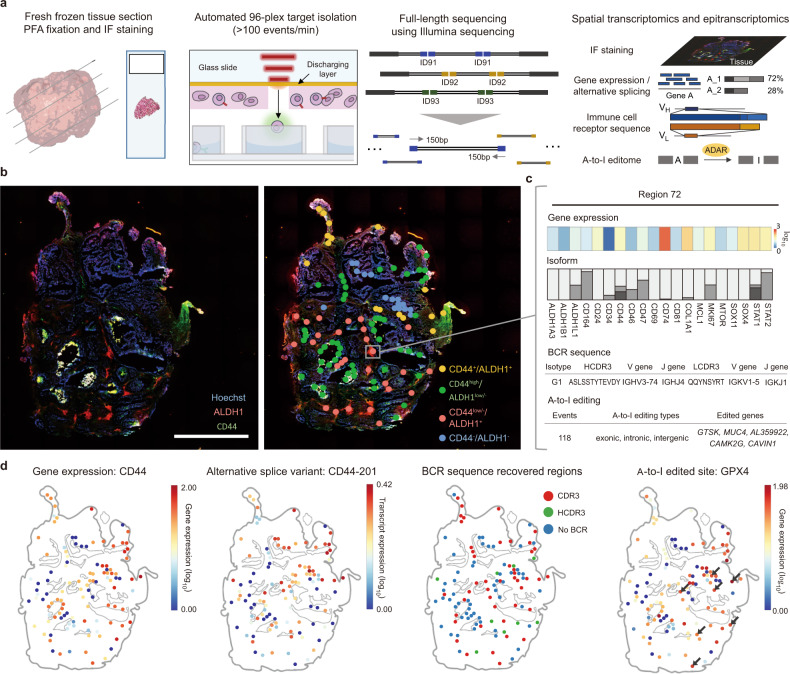
Spatial-histopathological examination-linked epitranscriptomics converged to transcriptomics with sequencing (Select-seq) enables full-length spatial transcriptomics and epitranscriptomics at single-nucleotide resolution. **a** Schematic of the Select-seq protocol. Selective isolation of target regions in tumour sections was performed using a near-infrared pulsed laser following immunofluorescence staining. Full-length transcripts extracted from each targeted region are tagged with barcodes used for tracking the target region. Multi-modal analysis of the full-length transcriptome is connected with the spatial and staining information using barcodes included in the sequencing data. **b** Targets selected from a triple-negative breast cancer (TNBC) patient tumour section. The tissue was stained with Hoechst dye and anti-ALDH1 and CD44 antibodies (scale bar, 100 μm). We obtained the above results from a single tissue section. **c** Transcriptomics and epitranscriptomics of target region 72 containing 5–30 cells at single-nucleotide resolution and its gene expression profiles, transcript isoforms, B cell receptor sequences, and adenosine-to-inosine (A-to-I) editing events. **d** Each transcriptomic and epitranscriptomic data point was mapped to the tissue based on the barcodes.

For the in-depth multi-modal analysis of the tumour microniches, we introduce spatial-histopathological examination-linked epitranscriptomics converged to transcriptomics with sequencing (Select-seq), a method that isolates regions of interest (ROIs) as small as single cells from immunofluorescence (IF)-stained tissue and obtains full-length transcriptome data at single-base resolution, connected to the spatial and staining information therein (Fig. 1a). Specifically, the selective isolation of every single ROI enables barcoding of the full portion of the transcriptome, which leads to an in-depth and multi-modal analysis of the tumour microniches. To address previous methodological difficulties, Select-seq was developed to isolate ROIs that contain a very small number of cells in high throughput (Supplementary Fig. 1). Leveraging the advantage of Select-seq, we investigated the hypothesis that cancer stem cells (CSCs) in triple-negative breast cancer (TNBC) have characteristic A-to-I editing-based regulation^17^. We explored the transcriptomic and epitranscriptomic landscape of TNBC tumours, whose microniches contain CSC-like cells. IF staining of two CSC-related proteins, CD44 and ALDH1, was applied to define the ROIs, which were selectively isolated using a pulsed near-infrared (NIR) laser retrieval system that isolates ~100 ROIs in 1 min^18^ (Fig. 1b). Then, we analyzed the transcriptomic and epitranscriptomic profiles in connection to the spatial and staining information for every ROI (Fig. 1c). We then spatially mapped gene expression, alternative splice variant expression, immune cell receptor sequences, and A-to-I-edited sequences (Fig. 1d). In the same microniches as well as microniches in other TNBC samples from additional four patients, we identified an A-to-I editome landscape. Especially, A-to-I-edited *GPX4* transcript (1106616) related to ferroptosis was identified in ALDH1-stained microniches. Together with the spatial transcriptomic data, the spatial A-to-I editome landscape will provide a deeper understanding of biological systems.

## Results

### Select-seq enables full-length spatial transcriptome analysis at the single-nucleotide level

**Fig. 2:**
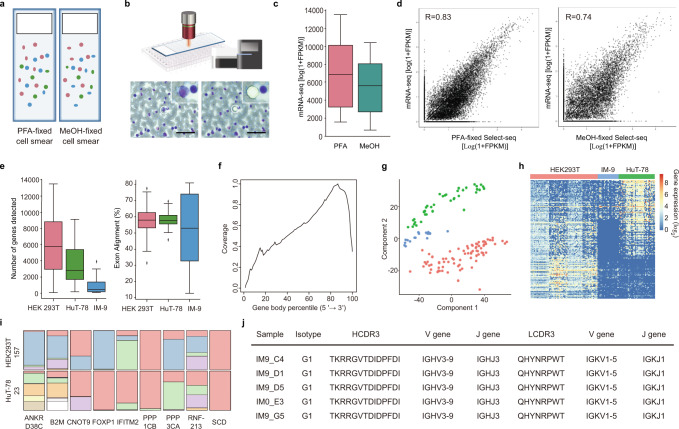
The Spatially-Resolved Laser-Activated Cell Sorting (SLACS) device produces high-quality spatial-histopathological examination-linked epitranscriptomics converged with transcriptomics with sequencing (Select-seq) data from single cells and ten cells. Source data are provided as a Source Data file. **a** Experimental design and an example of single-cell isolation. **b** Single-cell isolation of the SLACS device. Scale bar, 100 µm. **c** Fragments per kilobase of transcript per million mapped reads (FPKM) values for the paraformaldehyde (PFA)- and methanol (MeOH)-fixed cells (*n* = 60 biologically independent cells examined over 2 independent experiments). Interquartile range (IQR) of boxplot is between Q1 and Q3 and centre line indicates median value. Whiskers of boxplot is extended to the maxima and minima. Maxima is Q3 + 1.5*IQR and minima is Q1 − 1.5*IQR. **d** Correlation between the mRNA sequencing profiles from bulk mRNA-seq and Select-seq with ten types of PFA-fixed cells and Select-seq with ten types of MeOH-fixed cells. **e** Number of genes detected (FPKM) (left) and exon alignment percentage in three different cell lines fixed with PFA (right) (*n* = 92 biologically independent cells examined over 3 independent experiments). Interquartile range (IQR) of boxplot is between Q1 and Q3 and centre line indicates median value. Whiskers of boxplot is extended to the maxima and minima. Maxima is Q3 + 1.5*IQR and minima is Q1 − 1.5*IQR. **f** Representative 3’ end bias of the full-length transcriptomes. **g** Principal component analysis (PCA) and **h** unsupervised clustering heatmap of the cells analyzed with Select-seq. **i** Representative transcript isoform diversity from two samples. **j** B cell receptor (BCR) analysis of the IM-9 cell line.

To perform Select-seq, we cryosectioned fresh-frozen tissue samples with a thickness of ten micrometres and fixed them with paraformaldehyde (PFA). Then, with the in-house interface that marks the user-defined ROI (~5–10 cells) on top of the tissue image, the sections were subjected to pulsed laser-based ROI isolation into a retrieval PCR tube (Fig. 1a, b, and Supplementary Fig. 1). The optomechanical, non-contact isolation of cells guarantees intact nucleic acid for sequencing since NIR laser-induced vaporization of the transparent metal oxide layer deposited on a conventional glass slide^18,19^. For Select-seq, the retrieved ROIs were then lysed, and the molecules within were reverse-crosslinked^20^ for reverse transcription (RT) and PCR amplification^21^. Each isolated ROI was independently barcoded in a separate PCR tube and sequenced with the Illumina sequencing platform, generating full-length transcriptome data. From the full-length transcriptome data, we were able to multimodally analyze gene expression, mRNA alternative splice variants, immune cell receptor sequences, and A-to-I base editing events in relation to the spatial and immunofluorescence staining information (Fig. 1a). To validate Select-seq, we used three different cell lines of human origin (*n* = 152) (Fig. 2). The bulk RNA-seq data were more closely correlated with the laser-isolated PFA-fixed cell data (R = 0.83) than with the methanol-fixed cell data (R = 0.74). Additionally, we validated the qualities of the full-length transcriptome data attained by the procedures of fixed and recovered intact single-cell RNA (FRISCR)^20^. We examined the gene expression and alternatively spliced transcript profiles of three different cell lines as well as the sequences for T-cell receptors (TCRs) and B cell receptors (BCRs) in the HuT-78 and IM-9 cell lines, respectively. We compared fragments per kilobase of exon model per million reads mapped (FPKM) values of the unfixed and unstained cell with those of PFA-fixed cells and PFA-fixed stained cells. Because immune cells that comprise major parts of the tumour microenvironment are known to have low gene expression counts, we analyzed lymphocytic cell lines, IM-9 and HuT-78 cells. The medians for the number of detected genes were 4928, 730.75, and 2096 for HEK293T, IM-9, and HuT-78 cells, respectively (Fig. 2e). The exon alignment percentage was 52.80, 57.65, and 57.81% in the same order (Fig. 2e). The 5’end bias for the whole-transcriptome amplification process was measured (Fig. 2f). Furthermore, we determined that the different cell lines can be distinguished using the gene expression profiles (Fig. 2g, h). Finally, from the same full-length transcriptome data, alternatively spliced variant profiles (Fig. 2i) and BCR sequences (Fig. 2j) were recovered.

### Transcriptomic features of ALDH1^high^-stained regions with tumorigenicity

**Fig. 3:**
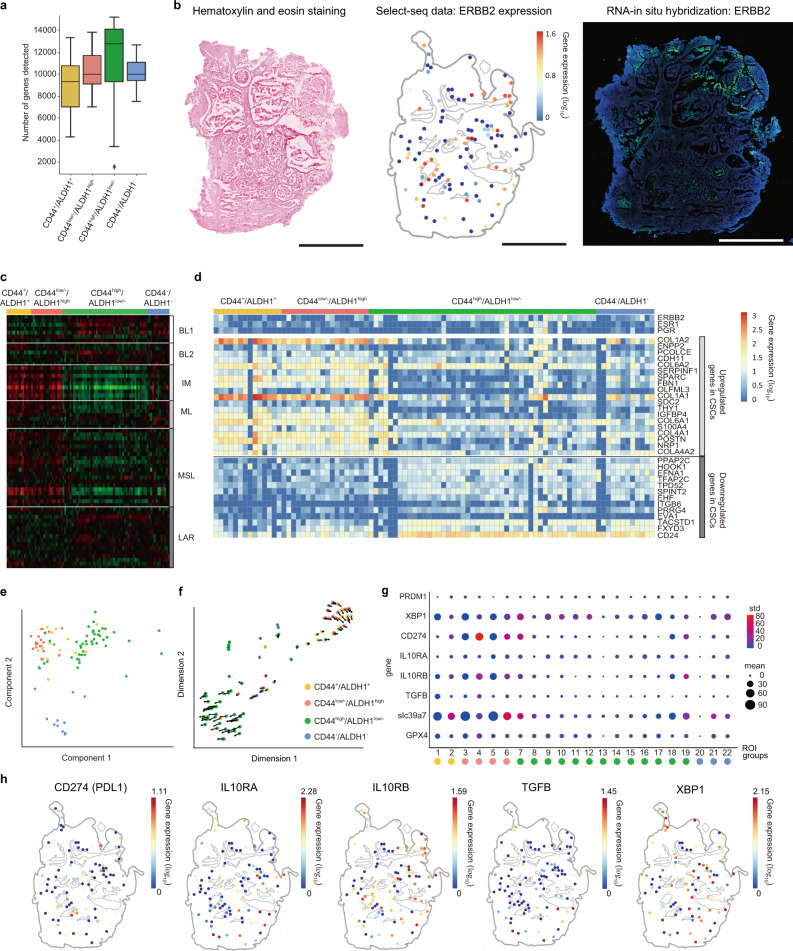
Tumour sections from TNBC patients reveal the spatial transcriptomic landscape of immunofluorescence (IF)-stained tissue sections. **a** Number of genes detected in the isolated target regions according to the four staining groups (*n* = 106 biologically independent samples (ROI)). Interquartile range (IQR) of boxplot is between Q1 and Q3 and centre line indicates median value. Whiskers of boxplot is extended to the maxima and minima. Maxima is Q3 + 1.5*IQR and minima is Q1 − 1.5*IQR. **b** Haematoxylin and eosin (H&E) stained serial tissue section (left). *ERBB2* gene expression data from Select-seq (middle). RNA-fluorescence in situ hybridization (FISH) of serial tissue sections (right) (scale bar, 500 μm). We obtained the above results from three different tissue sections. **c** Lehmann TNBC subtyping. Red and green boxes indicate upregulated and downregulated gene pathways. BL1 basal-like type 1, BL2 basal-like type 2, IM immunomodulatory, ML mesenchymal-like, MSL mesenchymal stem cell-like, LAR luminal androgen receptor. Source data are provided as a Source Data file. **d** Gene expression heatmap of the target regions. **e** Principal component analysis (PCA) of the target regions. **f** RNA velocity analysis of the target regions. Arrow indicates the A-to-I-edited sample in *GPX4*. **g** Mean and standard deviation (SD) of immunosuppressive gene signature and *GPX4* gene expression in different spatial groups. **h** Spatial mapping of signature genes related to immunosuppression. Yellow, green, red, and blue marks indicate (i) CD44^+^/ALDH1^+^, (ii) CD44^low/−^/ALDH1^high^, (iii) CD44^high^/ALDH1^low/−^, and (iv) CD44^−^/ALDH1^−^ regions, respectively, as determined by IF.

To investigate the spatial transcriptomic and epitranscriptomic landscape of TNBC, we performed Select-seq on five TNBC patients (Fig. 1 and Supplementary Fig. 2). To search for CSC-like microniches in a primary tumour, we initially investigated 106 target regions (Supplementary Fig. 3) in primary tumour sections from patient A (tissue A, ID: 190603). Fresh-frozen tissue sections were fixed with PFA and stained with IF probes targeting CD44 and ALDH1 (Fig. 1b) to determine the stem cell-like microniche within the tumour. We grouped the targets into four groups: (i) CD44^+^/ALDH1^+^, (ii) CD44^low/−^/ALDH1^high^, (iii) CD44^high^/ALDH1^low/−^, and (iv) CD44^−^/ALDH1^−^. The ROI groups were determined according to the presence of green fluorescence, red fluorescence, both, or neither. The quality of the full-length transcriptome was uniform when examined by the number of genes detected and the number of splice variants detected in the 106 ROIs (Fig. 3a and Supplementary Fig. 4). Then, in a consecutive slide, haematoxylin and eosin (H&E)-stained spatial features confirmed that the ROIs had histopathologically cancerous features (Fig. 3b). The gene expression levels of Erb-B2 receptor tyrosine kinase 2 (*ERBB2*) and *MKI67* from Select-seq were in agreement with the corresponding RNA in situ hybridization results in serial sections of the same tumour (Fig. 3b and Supplementary Fig. 3).

The microniches in the four staining groups with different immunofluorescence patterns were characterized according to the spatial transcriptomic data. We first analyzed the spatial heterogeneity of the tumour, revealing several Lehmann TNBC subtypes^22^ within the same tumour (Fig. 3c). The CD44^low/−^/ALDH1^high^ group and the CD44^+^/ALDH1^+^ group mostly consisted of immunomodulatory (IM) and mesenchymal stem cell-like (MSL) subtypes, while the CD44^high^/ALDH1^low/−^ group had a mixed population of basal-like-1 (BL1) TNBC subtypes. Although TNBC is a type of breast cancer that lacks oestrogen receptor (ER), progesterone receptor (PR), and ERBB2^22^ at the protein level, some populations within the tumour section expressed *ERBB2* at the gene level, suggesting the intratumoural heterogeneity of the breast cancer type within a given tumour (Fig. 3d, Supplementary data 1).

We also observed four different groups by principal component analysis (PCA) that were aligned to the immunofluorescence staining groups (Fig. 3e) except for the CD44^+^/ALDH1^+^ group that showed some overlap with The CD44^low/−^/ALDH1^high^ or CD44^high^/ALDH1^low/−^ group. To investigate the developmental relationship between the microniches, we examined the RNA velocity of the different microniches (Fig. 3f). The RNA velocity plot showed that the CD44^low/−^/ALDH1^high^ microniches tended to develop into CD44^high^/ALDH1^low/−^ microniches. Then, we analyzed whether the targets expressed previously reported cancer-related genes^23–26^. CSC gene expression signature patterns were observed mostly in the CD44^low/−^/ALDH1^high^ group and sometimes in the CD44^high^/ALDH1^low/−^ group (Fig. 3d). Furthermore, 22 previously reported upregulated genes in CSCs, such as *COL1A2*, *ENPP2,* and *PCOLCE*, and 15 downregulated genes, such as COL19A1, *PLPP2*, and *HOOK1*, were analyzed in the target ROIs^27^. The CD44^low/−^/ALDH1^high^ ROIs showed CSC-like gene expression features. When we analyzed the alternative splice variants, the biotype analysis from the ENSEMBL transcript reference showed that protein-coding alternative splice variants constituted 42.3% of all biotypes (Supplementary Fig. 4).

### Tumour infiltrating plasma cell gene signatures and corresponding BCR sequences are mapped onto the tumour microenvironment

**Fig. 4:**
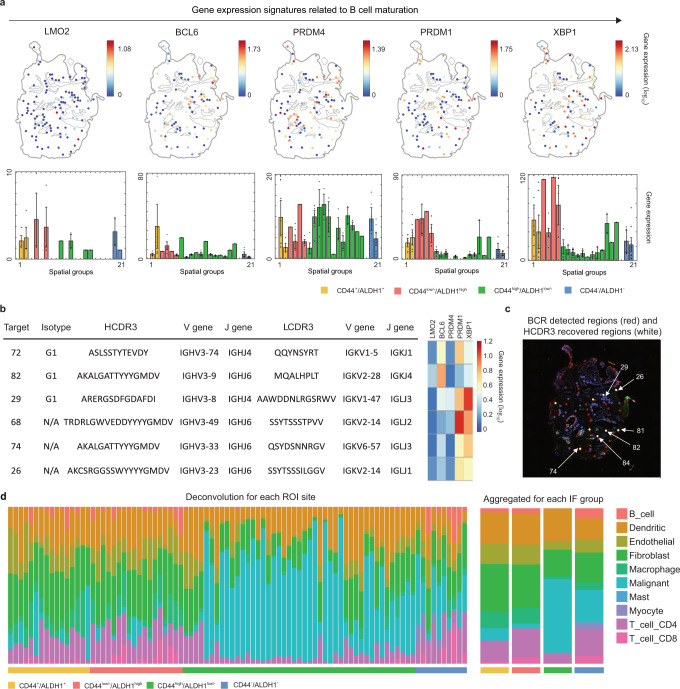
Spatial analysis of the immune cell repertoire. **a** Spatial mapping of the signature genes related to B cell maturation (*n* = 106 biologically independent samples (ROI)). Bar plot indicates mean value and error bar indicates standard deviation. **b** Six representative BCR sequences recovered using Select-seq and matched for gene expression related to B cell maturation. Overall, both heavy and light chain sequences from 42.9% of samples were recovered using Select-seq. Representative sequences including isotype, CDR3, matched V and J genes for each heavy and light chain. **c** Matched spatial context of recovered BCR sequences. Most of the BCRs were detected in the CD44low/−/ALDH1high stromal region, and very few BCRs were detected in the CD44high/ALDH1low/− duct region. Four of the six HCDR3 amino acid sequences were recovered from the CD44−/low/ALDH1high region, and two were recovered from the CD44−/ALDH1− region. **d** Single-cell deconvolution of each ROI (left) and ROI group (right). Source data for **b**–**d** are provided as a Source Data file.

To further assess the tumorigenicity of the CSC-like microniches, we examined the tumour immune microenvironment (Figs. 3g and 4). To effectively present gene expressions of ROIs that have the same staining phenotype and are close to each other, we grouped one to 12 ROIs and defined 22 ROI groups to compare the transcriptomic and epitranscriptomic information (Supplementary Table 2). We confirmed that immunomodulatory subtype-related gene ontology terms (Fig. 3c) and gene signatures related immunosuppression, such as PDL1 (*CD274*), interleukin 10 (*IL10RA, IL10RB*), TGF-β (*TGFB*), and XBP1, were observed in the CD44^low/−^/ALDH1^high^ ROI groups (Fig. 3g, h). We next examined the gene expression signatures for naïve B cells or centrocytes (LMO2 and BCL6), memory B cells (PRDM4), and plasmablasts or plasma cells (PRDM1 and XBP1)^28^ (Fig. 4a, b). It is interesting to note that the CD44^low/−^/ALDH1^high^ microniches where *PRDM1* and *XBP1* were highly expressed had high *IGHG1* expression indicating plasma cell infiltration that was suggested to be associated with poor prognosis^29^ (Fig. 4c). We were able to extract 7 TCRs and 204 BCRs from the same ROI data we used for gene expression typing analysis. Among them, CDR3 sequences were recovered from 74% of the total BCRs. The extracted BCR Heavy chain comprised of 25.7% G1 isotype and the isotypes for 74.3% were not recovered. Although additional data should be acquired, the prevalence of the HCDR3 amino acid sequence during gene rearrangement was confirmed^30^. To further characterize how these microniches are comprised of, we performed single-cell deconvolution using CIBERSORT^31^ (Fig. 4d). In the case of CD44-positive IF group, we observed the deconvolution result for each IF group and found out some sites were mainly composed of tumour cells and some other sites were composed of endothelial, fibroblast, and CD4 + T cells. These results were aligned with the tissue type we used and also showed the immune status of our tissue. Also, it is interesting to note that the cancer stem cell microniches with high ALDH1 expression seemed to be located more in the stromal region and through single-cell deconvolution of these microniches we were able to see that the fibroblast population is more observed in these regions. To view this trend at a glance, we brought the gene expression data of the ROIs together for each IF staining group and examined the distribution of different cell types. As we displayed B cell population existed in ROIs with high ALDH1 expression. Also, as discussed above, the fibroblast population seem to be larger in ALDH1 high expressing ROIs and a more malignant cell population is observed in CD44 high expressing ROIs.

### A-to-I editome specific to CD44^low/−^/ALDH1^high^-stained microniches is identified

**Fig. 5:**
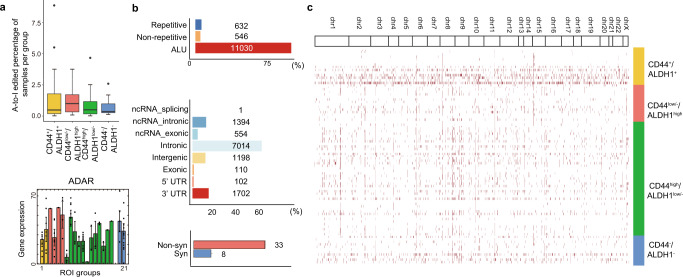
Spatial A-to-I editome marks characteristic features for different staining groups. Source data are provided as a Source Data file. **a** Percentage of A-to-I-edited samples per staining group (top) (*n* = 106 biologically independent samples (ROI)). Interquartile range (IQR) of boxplot is between Q1 and Q3 and centre line indicates median value. Whiskers of boxplot is extended to the maxima and minima. Maxima is Q3 + 1.5*IQR and minima is Q1 − 1.5*IQR. Bar graphs of *ADAR* gene expression according to the spatial groups (bottom). Bar plot indicates mean value and error bar indicates standard deviation. **b** Portions of A-to-I-edited regions in the genome (top). Profiles of editing in the non-repetitive regions, excluding upstream and downstream regions (middle). Portions of non-synonymous (Non-syn) and synonymous (Syn) editing events among the exonic-edited regions (bottom). **c** A-to-I editome landscape of different microniche groups.

**Fig. 6:**
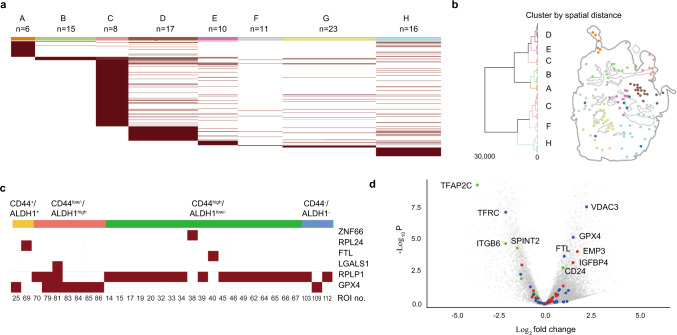
Non-synonymous A-to-I editing signatures are preserved in the stiaining microniche groups. Source data are provided as a Source Data file. **a** A-to-I editome of distant-clustered ROIs show preserved A-to-I signatures. **b** Tree with height clustered with physical distance between ROIs (left) and distribution of these clusters in the tissue. **c** A heatmap of single-base RNA editing event in selected genes related to iron mediation. **d** Volcano plot of fold change values of genes in samples with A-to-I-edited *GPX4* variants compared to those without the variant. Microniches from four TNBC tissues (from patients B, C, D, and E) were analyzed. Blue dots indicate ferroptosis-related genes, red dots indicate upregulated genes in CSCs, and green dots indicate downregulated genes in CSCs.

In addition to the transcriptomic landscape, we analyzed single-base resolution epitranscriptomics profiles to discover characteristic A-to-I-converted transcript variants in CSC-like microniches. This irreversible post-transcriptional deamination of adenosine to inosine in double-stranded RNA is catalyzed by the ADAR family^3,32,33^ and the converted inosine base is sequenced as a guanine base. The *ADAR* gene was uniformly expressed, implicating A-to-I events throughout the tissue (Fig. 5a and Supplementary Fig. 5). We observed a total of 12879 A-to-I-edited events using REDItools^34^ (Fig. 5b) in the first tissue and explored spatial a-to-I editome in the other four tissues (Supplementary Fig. 7). To validate the A-to-I events didn’t come from technical variation, we checked the correlation between the number of A-to-I events with the number of genes detected and *ADAR* gene expression. We found out the number of A-to-I events had a strong correlation with ADAR gene expression when grouped into four IF staining groups and had no meaningful correlation with the number of ADAR genes detected. Therefore, A-to-I-edited events we found throughout the tissue are meaningful RNA editing events (Supplementary Fig. 6). Among them, 632, 546, and 11,030 events were in repetitive, non-repetitive, and Alu regions in the genome, respectively. The exonic proportion of the edited sites was ~1%, and among them, non-synonymous editing consisted of 80% when annotated with the GENCODE reference^35^. Interestingly, A-to-I editome showed both shared and characteristic A-to-I-edited variants specific to the spatial groups categorized by IF staining and physical distance between the ROIs (Figs. 5c, 6a, b and Supplementary Data 2). We found out total 798 A-to-I editing events that were uniquely defined for each spatial group, which had differentially expressed genes (Supplementary Fig. 8). It is interesting to see not only that ROIs with shorter inter-distance show common A-to-I editome, but also that some genes like BRCA2, SOX9-AS1, and more are specific to spatial group C, which is a local mix of CD44^+^/ALDH1^+^ and CD44^low/−^/ALDH1^high^ cells in the stromal region between the two ducts with CD44^high^/ALDH1^low/−^ population. In terms of A-to-I editome specific to IF staining, characteristic non-synonymous A-to-I events were aligned to the *GPX4* gene at position 1106616 (rs713041), specifically in *GPX4* NM_001039848.4 alternative splice variants from the CD44^low/−^/ALDH1^high^ ROIs (Fig. 6c), with different frequencies ranging from 0.23 to 0.80, and were validated with capillary electrophoresis sequencing (Supplementary Fig. 5). A single-nucleotide polymorphism (SNP) has been reported in the same position (1106616)^36–38^, which was neither detected in the germline level nor tumour somatic variant in these cases, but study of the post-transcriptional variant has been limited. In the matched genomic DNA, we confirmed that only adenosine sequence is detected in the germline level nor tumour somatic variant in the corresponding position through Sanger sequencing and next-generation sequencing (Supplementary Fig. 9). Also, to assess if the corresponding position is theoretically accessible by the ADAR protein, we predicted the secondary structure with Forna^39^, and was confirmed to form double-stranded RNA in the site. The amino acid residue was altered from lysine residue to serine residue. Although the *GPX4* 203 variant we identified is reported to be a non-stop decaying mRNA, we assessed abnormalities that the *GPX4* transcript variant might have caused. We discovered that the CD44^low/−^/ALDH1^high^ microniches had exceptionally high gene set enrichment for ferroptosis, which is an emerging druggable target and can be further investigated by Select-seq (Supplementary Fig. 5). Other than this specific A-to-I variant, we were able to observe that more portions of ALU sites were edited from adenosine to inosine in ROIs with high ALDH1 expression. More A-to-I-edited ALU sites are closely related to worse patient outcomes^40^ and we suspect that this further suggests a potential epitranscriptomic signature specific to the ALDH1 high expressing cancer stem cell microniches.

### A-to-I editing events in GPX4 gene are related to ferroptosis-related gene expression

Ferroptosis is a non-apoptotic and lipid reactive oxygen species (ROS)-related type of programmed cell death that affects inflammation-associated immunosuppression in tumours^41,42^. We noted that the CD44^low/−^/ALDH1^high^ microniches with A-to-I-edited *GPX4* variants had high ferroptosis-associated gene set level changes with high expression levels of *GPX4*. With recent findings that breast tumours maintain a reservoir of subclonal diversity^43^, we sought to investigate whether the *GPX4* A-to-I editing event is related to residual cancer microniches. We applied Select-seq to four other patients who received neoadjuvant chemotherapy and supplied tissues B (ID: 180908T), C (ID: 180807T), D (ID: 190422T), and E (ID: 200710T), from which 161 target regions were selected for Select-seq (Supplementary Fig. 2). After screening the *GPX4* gene in the microniches in the four tissues, we separated them into two groups: those with the A-to-I-edited *GPX4* variant and those without the variant. We then depicted the volcano plot of the two different microniche groups in tissues B, C, D, and E (Fig. 6d). Specifically, the expression of the iron uptake protein-encoding gene *TFRC* was downregulated, while the expression of the iron storing ferritin-encoding gene *FTH1/FTL* and the lipid ROS elimination-related protein-encoding gene *GPX4* was upregulated. We suspect that similar to the previously reported A-to-C single-nucleotide variations at the genomic level, this alteration causes the overexpression of *GPX4* genes^36^. Additionally, pro-ferroptotic *VDAC3* gene expression in the A-to-I-edited microniches suggests that these cells will not develop resistance to ferroptosis-inducing drugs such as erastin, which binds to *VDAC3*^44^. In microniches without A-to-I editing in the *GPX4* gene, the *TFAP2C*, *ITGB6*, and *SPINT2* genes, which are known to be downregulated in CSCs, were overexpressed. Therefore, differentially expressed ferroptosis-related genes and *GPX4* A-to-I editing events throughout the tissue were observed and led to our hypothesis that *GPX4* A-to-I editing events affect ferroptosis in CSC-like microniches. We further explored whether A-to-I editing in *GPX4* genes was related to clinical outcomes in TNBC patients.

We sought to analyze other bulk TNBC transcriptomic datasets that are publicly available in TCGA to see the relation. Bulk transcriptome data from 109 TNBC patients were analyzed, 80 of which patients had the GPX4 variants that included A-to-I-edited variants, SNPs or somatic mutations at the genome level. We observed the gene set related to ferroptosis was highly enriched when the median frequency of the GPX4 variant in the transcript data was higher (Supplementary Fig. 10). The overall survival rates of the two groups estimated using the Kaplan–Meier method showed that the group with a high frequency of the GPX4 1106616 variant at the transcriptome level (positive ferroptosis gene set enrichment value) showed a trend toward worse clinical outcomes compared to the group with a low frequency of the variant (Supplementary Fig. 10). Furthermore, we compared the correlation of survival with a combination of *ADAR* genes, total A-to-I events, and even patients who have SNP within GPX4. We found out none of them showed similar results but only GPX4 1106616 variant has the most strong correlation with survival difference grouped by ferroptosis gene set enrichment value (Supplementary Fig. 11). Although more assessments are required to validate that targeting GPX4 abnormalities would provide therapeutic responses, Select-seq was able to unveil GPX4 A-to-I editing events in relation to CSC-like gene expression signatures in TNBC patients.

## Discussion

Using Select-seq, we revealed that the CD44^low/−^/ALDH1^high^ microniches in the stromal regions had transcriptomic signatures with high levels of B cell activity and A-to-I editing. Furthermore, we were able to efficiently find the link between the epitranscriptomics signatures of CSC-like microniches in TNBC tumours. In particular, we identified *GPX4* A-to-I variants related to clinical outcomes. Although the single-base RNA edited variants should further be assessed with additional experiments such as applying recombinant ADAR to the variant in the future or methodologies like cyanoethylation and RT-PCR sequencing^45^, these findings can provide clues to identify useful CSC-related druggable targets as emerging therapeutic targets^46^. With ROI-based, in-depth and multi-modal analysis, Select-seq allows comprehensive spatial epitranscriptomic analysis. Although we could match only a few immune cell receptors of the tumour infiltrating lymphocytes to the existing database, further studies that can pair the antigen-expressing genes of these receptors will lead to deeper studies of how these tissue infiltrating immune cells are interacting in the cancer microenvironment. Also, the studies using alternative splicing variants can be extended to study fusion genes for potential drug targets. By integrating the current state-of-art highly parallel spatial transcriptome^47,48^, single-cell RNA sequencing^15,49^, and single-cell deconvolution^15,50^ technologies, Select-seq can reveal more complex mechanisms that do not solely depend on the gene expression profiles. Furthermore, by adding modalities such as DNA sequencing, mass spectrometry, or other image-based analysis techniques such as in situ sequencing to the spatial transcriptome data in target regions, Select-seq will become a complementary tool that enables the deep analysis of ROIs.

## Methods

### Region of interest (ROI) isolation

The Spatially-resolved Laser Activated Cell Sorter (SLACS) instrument comprises optical modules and mechanical modules for high-throughput retrieval of samples from the tissue. Two motorized stages exist for handling the retrieved targets. An *X-Y* axis motorized stage (ACS Motion Control, Migdal, Israel) was built to control the spatial location of the target. The device can be controlled automatically by communicating with a computer. One stage is for loading sample slides, and the other stage is for loading tubes to receive isolated cells. A charge-coupled device (CCD) camera (Jenoptik, Jena, Germany) was installed to observe where the laser pulse will be applied through the objective lenses. A neodymium-doped yttrium aluminum garnet (Nd:YAG) nanosecond laser was purchased from Continuum (Minilite™ Series ML II; Continuum, San Jose, CA). There is a slit located in the light path between the laser source and the objective lens to control the region to be isolated. The slit is controlled either manually or automatically to adjust the size of the laser pulse. Objective lenses with various magnifications were purchased from Mitutoyo. The long working distance allows more space between the lens and the sample for user convenience.

### Communication system for SLACS

We designed two different pieces of software, which were written in Python scripts. The first was built for the user (a pathologist in our case) to select the cells to be isolated. We shared the whole-slide image with the user through a server, and the user ran the software to select the cells of interest while navigating the tissue image through the graphical user interface. After selection, the program produces two files: a text file with locational information about the region of interest and the image file with the selected targets is overlaid with transparent blue on the original image. Both files are required for the automated isolation of the target cells. The second software enables the automatic control of the SLACS instrument. With this software, the users are able to control the slits, change the objective lenses, and move the motorized stages. It also enables automatic target isolation when two files from the first software are loaded. All tissue samples were isolated using an automatic function, while the cell line experiments were performed manually.

### Cell culture

Human HEK 293 T (cat # 21573), human IM-9 cells (cat # 10159), human HuT-78 cells (cat # 90078), and murine NIH3T3 cells (cat # 21658) were purchased from Korean Cell Line Bank (KCLB) and propagated according to the manufacturer’s instructions. HEK 293 T cells were cultured in DMEM (Thermo Fischer Scientific, Massachusetts, USA) with 1% penicillin-streptomycin (Corning, New York, USA) and 10% foetal bovine serum (HyClone, Massachusetts, USA) at 37 °C under 5% CO2 and 95% atmospheric air; IM-9 cells were cultured in RPMI 1640 (Thermo Fischer Scientific) with 1% penicillin-streptomycin (Corning, New York, USA) and 10% foetal bovine serum (FBS, HyClone) at 37 °C under 5% CO2 and 95% atmospheric air; HuT cells were cultured in DMEM (Thermo Fischer Scientific, Massachusetts, USA) with 1% penicillin-streptomycin (Corning, New York, USA) and 10% FBS (HyClone, Massachusetts, USA) at 37 °C under 5% CO2 and 95% atmospheric air. NIH3T3 cells were cultured in DMEM (Thermo Fischer Scientific) with 1% penicillin-streptomycin (Corning, New York, USA) and 10% bovine calf serum (HyClone) at 37 °C under 5% CO_2_ and 95% atmospheric air. Adherent cells such as HEK 293 T cells and NIH3T3 cells were grown to a confluence of 50–80% and treated with TrypLE (Invitrogen, California, USA) for five min, quenched with an equal volume of the growth medium, and spun down at 1500 rpm for 3 min. In addition, suspension cells such as IM-9 and HuT-78 cells were grown to a concentration of 2 × 10^5^–5 × 10^5^ cells/mL and spun down at 1500 rpm for 3 min. Then, the supernatant was removed, and cells were resuspended in 1 mL of 1× PBS with 10 μl RNase Inhibitor (Invitrogen, California, USA) and re-spun at 1500 rpm for 3 min. The supernatant was again removed, and cells were resuspended in 10 μl and spread on indium tin oxide (ITO) glass (Fine Chemicals Industry, Republic of Korea).

For paraformaldehyde (PFA) fixation, smeared cells were fixed with 4% PFA solution (Thermo Fischer Scientific, Massachusetts, USA) at 4 °C for 15 min and washed with DPBS at room temperature; for methanol (MeOH) fixation, smeared cells were serially fixed with 70%, 90%, and 99% MeOH in distilled water for 30 s and rehydrated by dipping for 30 s in reverse order.

### Tissue preparation

Tissue sections were acquired from the archives of the biorepository of Lab of Breast Cancer Biology at the Cancer Research Institute, Seoul National University. The preparation of tissue was approved by the Institutional Review Board of Seoul National University Hospital (SNUH, IRB No. 1405-088-580). The patients provided written consent without compensation for the archiving and use of tissue and blood samples for research purposes. For PFA fixation, we fixed the tissue sections with 4% PFA in PBS on ice for 15 min and then washed them twice with ice-cold PBS containing 1% recombinant RNase inhibitor (Takara, Japan). The section was air-dried on ice for 3 min. For MeOH fixation, we fixed the tissue section in rising methanol concentrations (75, 95, and 99% MeOH, 30 s each) and air-dried them on ice for 1 min.

For haematoxylin and eosin (H&E) staining, we fixed the tissue sections with 4% PFA as described above, except for the air-drying process. Then, the tissue sections were stained with Mayer’s haematoxylin (Sigma–Aldrich, Germany) for 1 min and bluing buffer (Agilent Dako, US, California) for 30 s followed by eosin (Sigma–Aldrich, Germany) for 10 s. We washed the sample with distilled pure water for 30 s between each buffer changing process during staining. Finally, we dipped the sample in 70% MeOH for 30 s and air-dried the sample on ice for 1 min. For immunofluorescence staining, we fixed the tissue section with 4% PFA as described above, except for the air-drying process. Then, we blocked the sections in PBS with 1% BSA for 15 min on ice. We diluted primary antibodies (1:200) in blocking solution, incubated the slides with primary antibodies for 15 min on ice, and then washed them twice with ice-cold PBS. The tissue sections were air-dried for 3 min on ice, and fluorescence images were acquired on a microscope (Nikon Eclipse Ti). We used primary antibodies against CD4 (Abcam, UK, ab181724), CD8 (Abcam, UK, ab251596), CD14 (Abcam, UK, ab230903), and CD19 (Abcam, UK, ab237772). We used the following fluorescence conjugate kits for each antibody: Alexa 488 (Abcam, UK, ab236553), TxRed (Abcam, UK, ab195225), Cy3 (Abcam, UK, ab188287), and Cy5 (Abcam, UK, ab188288).

### Modified Smart-seq2

To lyse cells, isolated cells were added to a 0.2 ml thin-wall PCR tube containing 2 μl of a mild hypotonic lysis buffer composed of 0.2% Triton X-100 (Sigma–Aldrich, Germany) and 2U/μl of recombinant RNase inhibitor (40 U/μl, Takara, Japan), 1 μl of 10 mM oligo-dT primer (Macrogen, Republic of Korea, 5’- AAGCAGTGGTATCAACGCAGAGTACT_30_VN-3’), and 1 μl of 10 mM dNTP mix (Takara, Japan) and were incubated at 72 °C for 15 min and then immediately placed on iced. In the case of PFA-fixed samples, fixed cells were added to 0.2 ml thin-wall PCR tubes containing 2 μl of an enzymatic lysis buffer composed of 2.5 mg/ml Proteinase K (10 mg/ml, Sigma–Aldrich, Germany) in nuclease-free water (Invitrogen, California, USA), 1 μl of oligo-dT primer, and 1 μl of dNTP mix (Takara, Japan), and then they were incubated at 50 °C for 1 h^20^, 70 °C for 10 min, and immediately placed on iced afterwards.

cDNA obtained from extracted RNA was prepared with SMART-seq2 protocol^21^ with the following modifications: we prepared 6 μl of the first-strand reaction mix, containing 0.5 μl SuperScript II (200 U/μl, Invitrogen, California, USA), 0.25 μl recombinant RNase inhibitor (40 U/μl, Takara, Japan) 2 μl SuperScript II First-Strand Buffer (5×, Invitrogen, California, USA), 0.25 μl dichlorodiphenyltrichloroethane (DTT) (100 mM, Invitrogen, California, USA), 2 μl betaine (5 M, Sigma–Aldrich, Germany), 0.06 μl MgCl_2_ (1 M, Sigma–Aldrich, Germany), 0.1 μl template switching oligo (TSO) (100 μM, Bioneer, Republic of Korea, 5′-AAGCAGTGGTATCAACGCAGAGTACrGrG+G-3′), and 0.59 μl nuclease-free water (Qiagen, Germany).

After reverse transcription and template switching, cDNA was amplified with an additional PCR mix composed of 12.5 μl of KAPA HotStart HIFI 2× Ready Mix (Kapa Biosystems, Switzerland), 0.25 μl of PCR primers (10 μM, Macrogen, Republic of Korea, 5′-AAGCAGTGGTATCAACGCAGAGT-3′), and 2.25 μl of nuclease-free water (Qiagen, Germany) for 20 or 25 cycles for RNA from single cells^21^. PCR products were purified using CeleMag beads (Celemics, Republic of Korea). The average fragment length of purified cDNA was determined by electrophoresis within a 1.2% agarose gel, and the concentration of cDNA was determined using the Qubit dsDNA High Sensitivity Assay Kit (Life Technologies, California, USA) according to the manufacturer’s protocol.

### Next-generation sequencing

The pTXB1 cloning vector, which introduced hyperactive E54K and L372P mutations into wild-type Tn5, was acquired from Addgene. pTXB1 Tn5 and its mutants were expressed and purified^51^. Then, 50 bp paired-end sequencing was performed on an Illumina NextSeq sequencing platform, resulting in an average read depth of ~600 M reads per sample.

### Read alignment and gene quantification

We demultiplexed and trimmed the raw sequencing reads using Cutadapt^52^. Then, we filtered out the reads with a sequencing quality less than 15 and a read length shorter than 25 bp. The remaining reads were aligned against the mouse genome (GRCm38) for mouse cell line samples and against the human genome (GRCh38) for human cell line samples and breast cancer samples using STAR aligner^53^ with the default settings. The number of uniquely mapped reads of each sample was calculated using featureCounts with default parameters for downstream differential expression analysis and RNA-seq by expectation-maximization (RSEM) with default parameters for fragments per kilobase of transcript per million mapped reads (FPKM) count. To quantify and normalize the expression of genes, data from FeatureCounts^54^ were normalized using the normalization method implemented in DESeq2^55^.

### Spatial gene expression visualization

The spatial gene expression was plotted in Python and matplotlib using the gene expression data from normalized FeatureCounts and matched positional information data of isolated samples that were selected and recorded by custom software before the isolation process.

### Differential gene expression analysis

For analysis of differential gene expression between samples or selected spatial regions, we conducted differential gene expression analysis using DESeq2. Only genes with a log2-fold change >1 and an adjusted *p*-value of <0.05 were considered positive differentially expressed genes.

Pathway analysis was performed using the R package gage^56^ on the normalized expression counts using DESeq2, and the mapped pathway was visualized using the R package pathview^57^. Gene expression levels were mapped to corresponding pathways by Kyoto Encyclopedia of Gene and Genomes (KEGG) enrichment or Molecular Signatures Database (MSigDB: http://www.broad.mit.edu/gsea) analysis. The optimal number of stable triple-negative breast cancer (TNBC) subtypes was determined by using the lists of gene sets followed by Lehmann TNBC subtypes.

### Fluorescence in situ hybridization (FISH)

Stellaris® FISH RNA Probes (Human ERBB2 with Quasar® 570 Dye) and the other reagents were purchased from Biosearch Technologies (California, USA). Fresh-frozen dissected tissues at a thickness of 4–10 μm were mounted onto microscope slides. The slide-mounted tissue sections were immersed in fixation buffer (3.7% formaldehyde in RNase-free PBS) for 10 min at room temperature, washed twice with PBS for 5 min, permeabilized with 70% ethanol for 1 h at room temperature and immersed in wash buffer A for 5 min. The tissue sections were incubated with a hybridization buffer containing the probe at 37 °C for 4 h followed by counterstaining the nuclei with wash buffer A consisting of 5 ng/ml DAPI. The slides were rinsed with wash buffer B and mounted with an aqueous mounting medium.

### RNA velocity analyses

RNA velocity was calculated on the basis of spliced and unspliced transcript reads. Based on the velocyto pipeline, annotation of spliced and unspliced reads was performed using the Python script velocyto.py and run_smartseq2 options. Annotated reads were filtered based on the number of reads of exonic, intronic, and spanning regions. Principal component analysis (PCA) and t-distributed stochastic neighbour embedding (t-SNE) analysis were performed using filtered reads, and RNA velocity was estimated using a gene-relative model with k-nearest neighbour cell pooling (k = 10). Velocity was projected onto the embedding space of PCA or t-SNE. We used the standard R implementation of velocyto default settings with velocyto.R.

### RNA editing analysis by REDItools

RNA editing sites were detected from STAR-aligned reads using REDItools^34^. Harsh parameter settings (-c 10,10 -m 25,25 -v 3 -q 25,25 -e -n 0.1 -u -l -p) were applied for calling RNA editing events to reduce false positives. A list of known adenosine-to-inosine (A-to-I) editing sites was downloaded from the REDIportal database, and only the known editing sites were selected. In total, 20,367 editing sites were detected and applied to further A-to-I editing analysis.

### TCR and BCR extraction

The TCR sequences and BCR sequences for each sample were assembled using TraCeR^58^ and BraCeR^59^, which allowed the reconstruction of the TCR and BCR sequences from the RNA-seq data. With both software programs, we obtained the CDR3 sequence and the rearranged TCR and BCR genes from the sample that we spatially isolated. TraCeR and BraCeR both first made the reference file that has the possible combination of V and J genes. Then, RNA-seq reads from each cell were aligned against each reference file using the Bowtie2 aligner. The reads that aligned to the appropriate reference were assembled by Trinity RNA-seq assembly software. Finally, contigs assembled by Trinity were used as input to IgBlast, and the resulting output text files provided information about the CDR3 sequence and the rearranged TCR and BCR genes. To display rearranged TCR and BCR genes, we first filtered only those genes whose E value of the V gene was 5*10^−3 or less. Then, we reconstructed the genes with the V genes, D genes, and J genes that had the highest E value. The CDR3 region is displayed if the IgBlast output text file gives us information about the CDR3 region. We filtered the sequences with stop codons and poly A tail sequences in the end sequence. If there were more than three matching genes, we displayed the first three genes from IgBlast.

### CIBERSORT

The cell type proportions in the different samples were then estimated using cibersortx^31^, which detects cell-type-specific signature genes using annotated single-cell data. As input, we used the number of uniquely mapped reads of each sample from featureCounts. Cibersortx needs a signature matrix, so first, we used the NSCLC PBMCs Single Cell RNA-Seq^31^ signature matrix which is online. Calculating cell type proportions was performed with the default parameter.

### Public data processing

We obtained an additional 115 TNBC aligned RNA-seq files from TCGA^60^ via the Genomic Data Commons Data Portal. The clinical data corresponding to each sample were obtained for survival analysis. The number of uniquely mapped reads of each sample was calculated using featureCounts with the same setting we used. Then, pathway analysis related to ferroptosis (hsa04216) was performed on each sample compared to the remaining 109 samples except for 6 samples from normal tissues. Samples with a positive number of statistics, in which ferroptosis-related genes were expressed more than other samples, were grouped as upregulated; those with a negative number of statistics were grouped as downregulated and those that were not grouped were divided into a negative group. The upregulated and downregulated groups included 70 and 39 samples, respectively. The density of frequency of A-to-I editing events in each group was expressed through a violin plot, and the black square indicates the median value of frequency. Based on these groups, we conducted a survival analysis using the Lifelines python package (version 0.25.7)^61^. We conducted Kaplan–Meier analysis comparing the upregulated and downregulated groups, excluding those with <30 days of follow-up. Survival curves for the two groups were compared using the log-rank test (lifelines.statistics.logrank_test).

### Reporting summary

Further information on research design is available in the Nature Research Reporting Summary linked to this article.

## Supplementary information

Supplementary Information

Dataset1

Dataset2

Reporting Summary

## Data Availability

The raw sequencing data files generated in this study have been deposited in the Sequence Read Archive as processed bam file under the bioproject code PRJNA779567. Readers can freely access the uploaded datasets through Sequence Read Archive. The TCGA gene expression data and patient’s metadata are available from NIH genomic data commons (https://portal.gdc.cancer.gov/projects/TCGA-BRCA). We also used Molecular Signatures Database (MSigDB) (https://www.gsea-msigdb.org/gsea/msigdb/), Kyoto Encyclopedia of Gene and Genomes (KEGG) (https://www.genome.jp/kegg/), and REDIportal database (http://srv00.recas.ba.infn.it/atlas/) for our analysis. The remaining data are available within the Article (Differentially expressed genes within tissue A, Position of editied GPX4), Supplementary information (Gene expression, A-to-I editing, spatial group of tissue A), or Source Data file (Gene expression and immune cell receptor of cell line; Gene ontology enrichment value and A-to-I editing of entire tissues; Immune cell receptor, cell type composition, transcript quantification, and A-to-I heatmap value of tissue A; GPX4 A-to-I editing of TCGA-BRCA). Source data are provided with this paper.

## Source data

Source Data

## Code availability

Source code is available at GitHub (https://github.com/BiNEL-SNU/Select-seq)^62^.
