# Supplementary material for: Spatial epitranscriptomics reveals A-to-I editome specific to cancer stem cell microniches
Source: Nat Commun. 2022 May 9;13:2540. doi: 10.1038/s41467-022-30299-3 (PMC9085828; doi:10.1038/s41467-022-30299-3)
Supplement: Supplementary file 1 — Supplementary Information [file 41467_2022_30299_MOESM1_ESM.pdf]

Supplementary materials

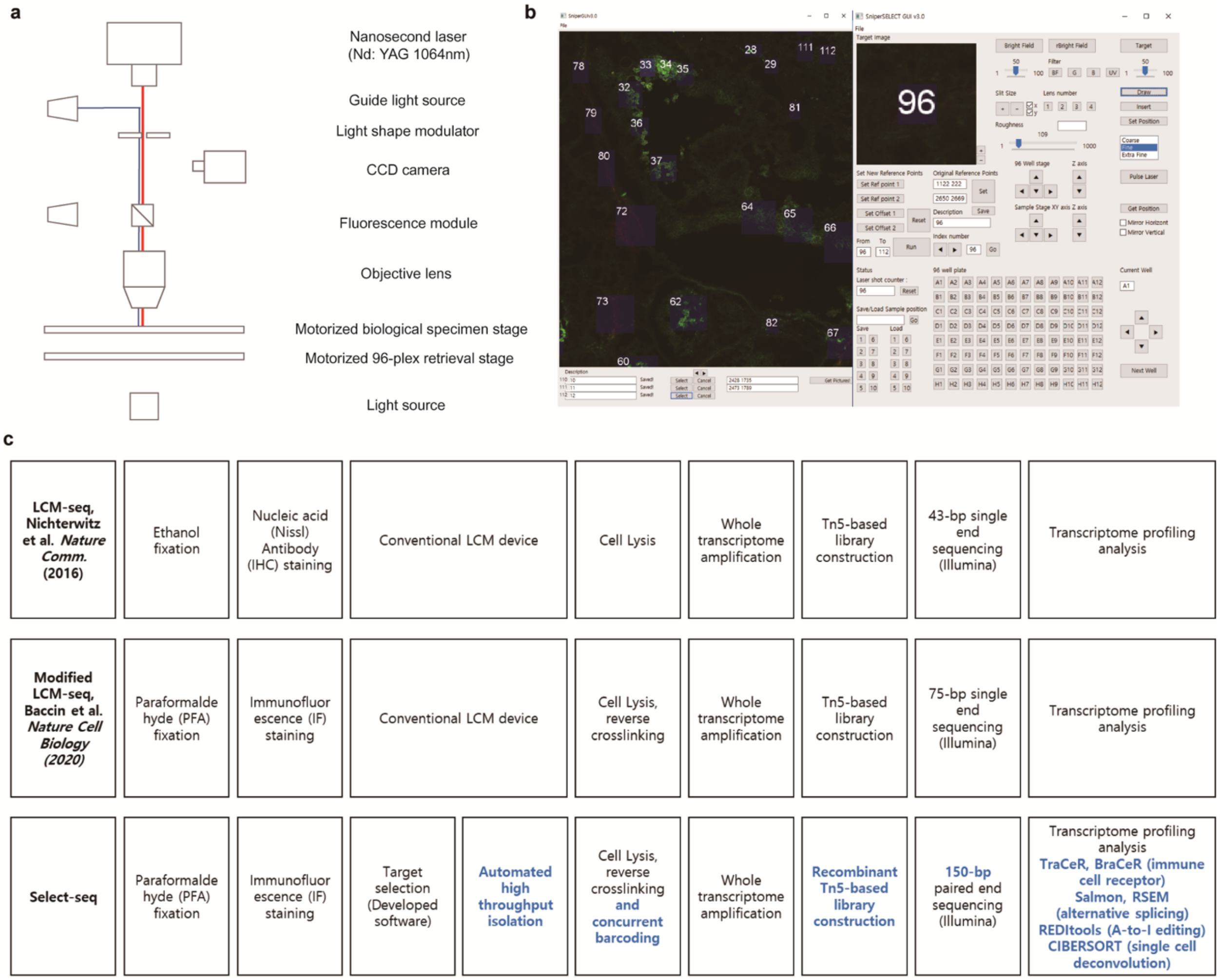

Supplementary Fig. 1. The Spatially-Resolved Laser-Activated Cell Sorting (SLACS) device. a, The SLACS device. **b,** Software for automatic isolation of the target regions of interest (ROIs). **c,** Comparison of Select-seq to previous methodologies that utilize laser capture microdissection.

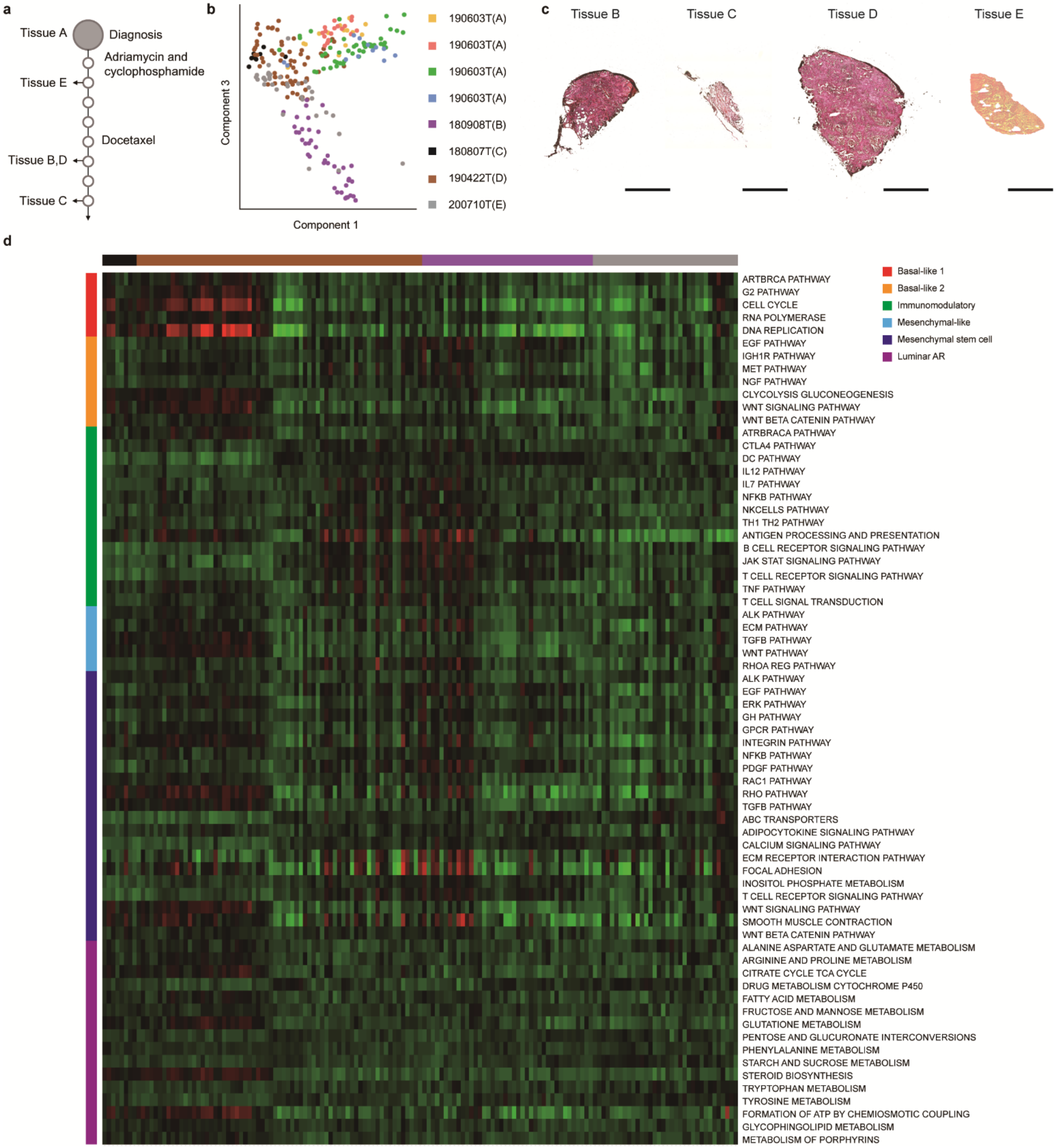

Supplementary Fig. 2. Image data of tissues from the five triple-negative breast cancer (TNBC) patients. Each tissue was stained using fluorophore-labelled antibodies to identify target cells, and the whole slides were scanned. **a,** Different clinical conditions for the five

TNBC patients. **b,** PCA of the transcriptome data from the ROIs from the five TNBC patients. **c,** Hematoxylin and eosin (H&E) images for four other tissues from patients B, C, D and E. Scale bar, 500 µm. **d,** Gene set enrichment patterns according to Lehmann TNBC subtype in patients B, C, D, and E. Rows indicate the mean selected pathways based on Lehmann TNBC classification, and columns represent the total TNBC samples. Source data are provided as a Source Data file.

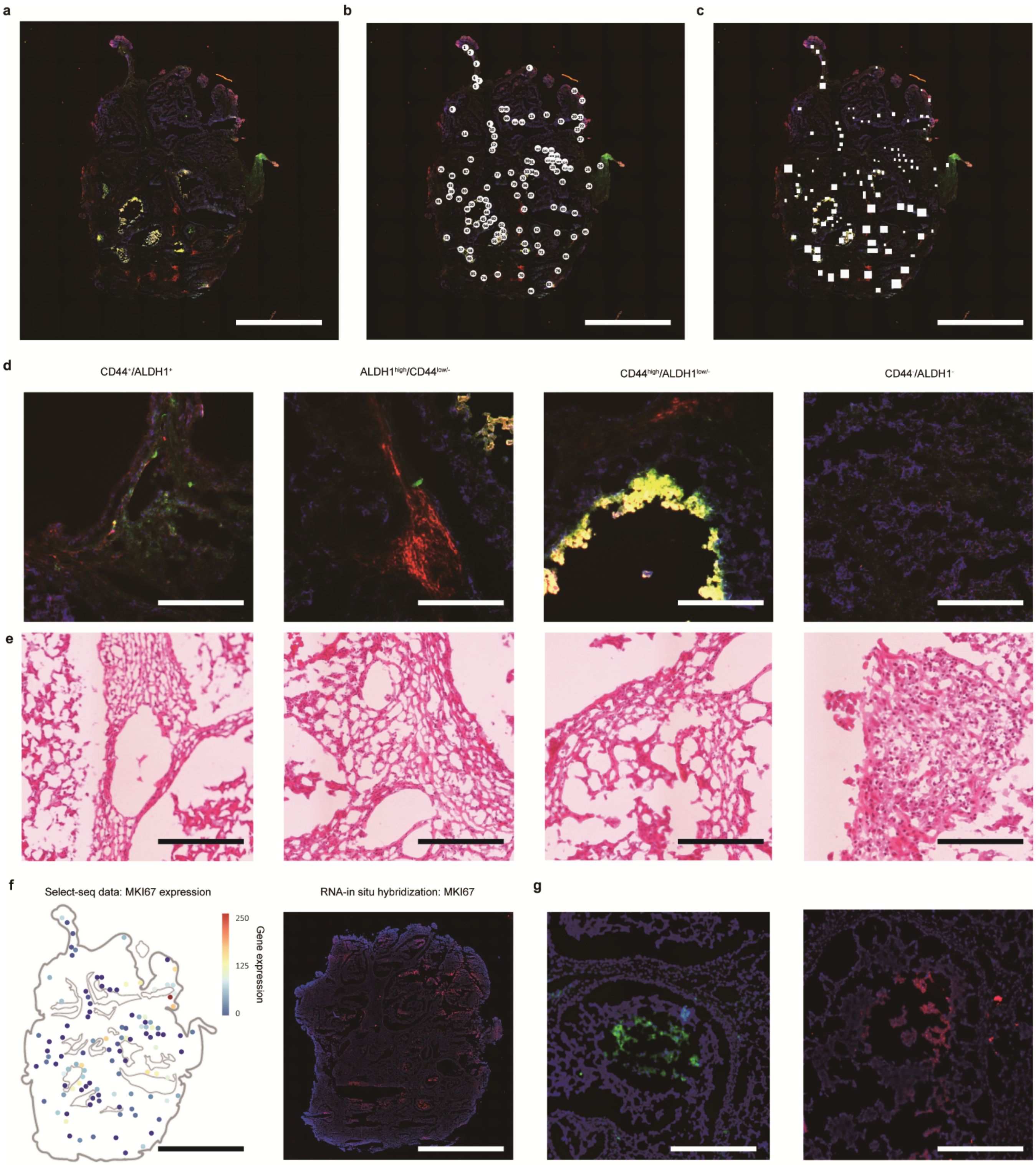

Supplementary Fig. 3. Image data of tissues from patient. Each tissue was stained using fluorophore-labelled antibodies to identify target cells, and the whole slides were scanned.

Results of **a-d** were obtained from the same tissue and **g** was obtained from a adjacent tissue of **a-d**. **a,** Immunofluorescently stained image of tissue A. Scale bar, 500 µm. **b,** The numbering for the target ROIs from the same tissue. Scale bar, 500 µm. **c,** Target region area on the same tissue. Scale bar, 500 µm. **d,** Zoomed-in images of the 4 different staining groups. Scale bar, 100 µm. **e,** Corresponding H&E images for the zoomed-in images presented in d. Scale bar, 100 µm. **f,** Different clinical conditions for the five TNBC patients. Scale bar, 500 µm. **g,** H&E images for other four tissues. Scale bar, 100 µm.

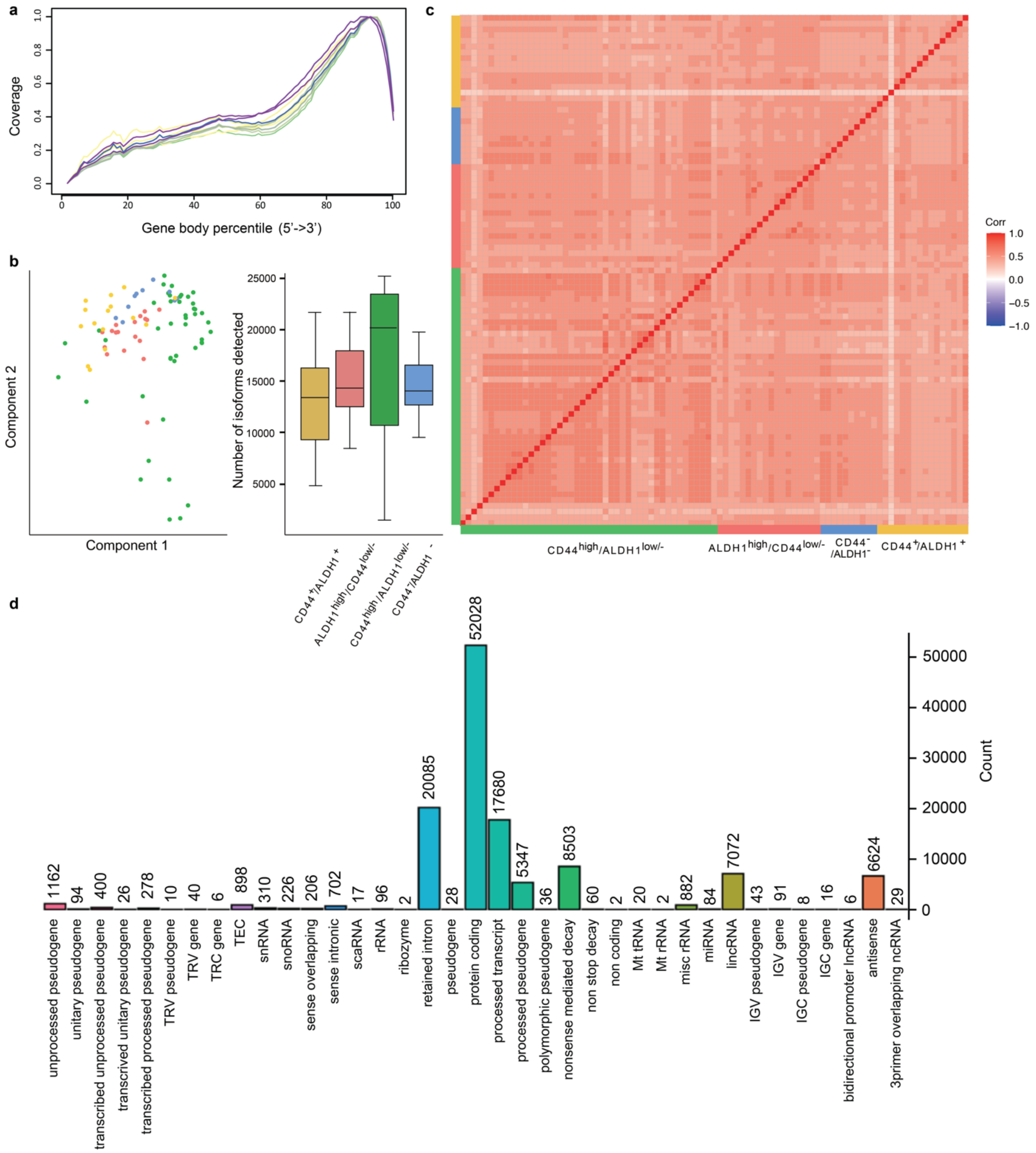

Supplementary Fig. 4. Select-seq allows analysis of alternative splicing variants. Source data are provided as a Source Data file. **a,** Gene body coverage of isolated regions of tissue A with Select-seq. We obtained sufficient coverage to reach the isoform resolution even though the samples were fixed with PFA. **b,** PCA and number of detected isoforms in each staining

modality group (n=106 biologically independent samples (ROI)). Interquartile range (IQR) of box plot is between Q1 – Q3 and centre line indicates median value. Whiskers of box plot is extended to the maxima and minima. Maxima is Q3 + 1.5*IQR and minima is Q1 – 1.5*IQR. The staining groups formed more distinct groups when clustered by gene counts than when clustered by transcripts. The median numbers of detected transcripts for the staining groups were 13,833, 14,674, 20,711 and 14,580. **c,** Correlation analysis of each staining modality group using transcripts. **d,** The number of transcripts in each functional annotation group. Select-seq recovered the full-length transcriptome and thus obtained various functionally different isoforms, including immune receptor-related isoforms and noncoding RNAs.

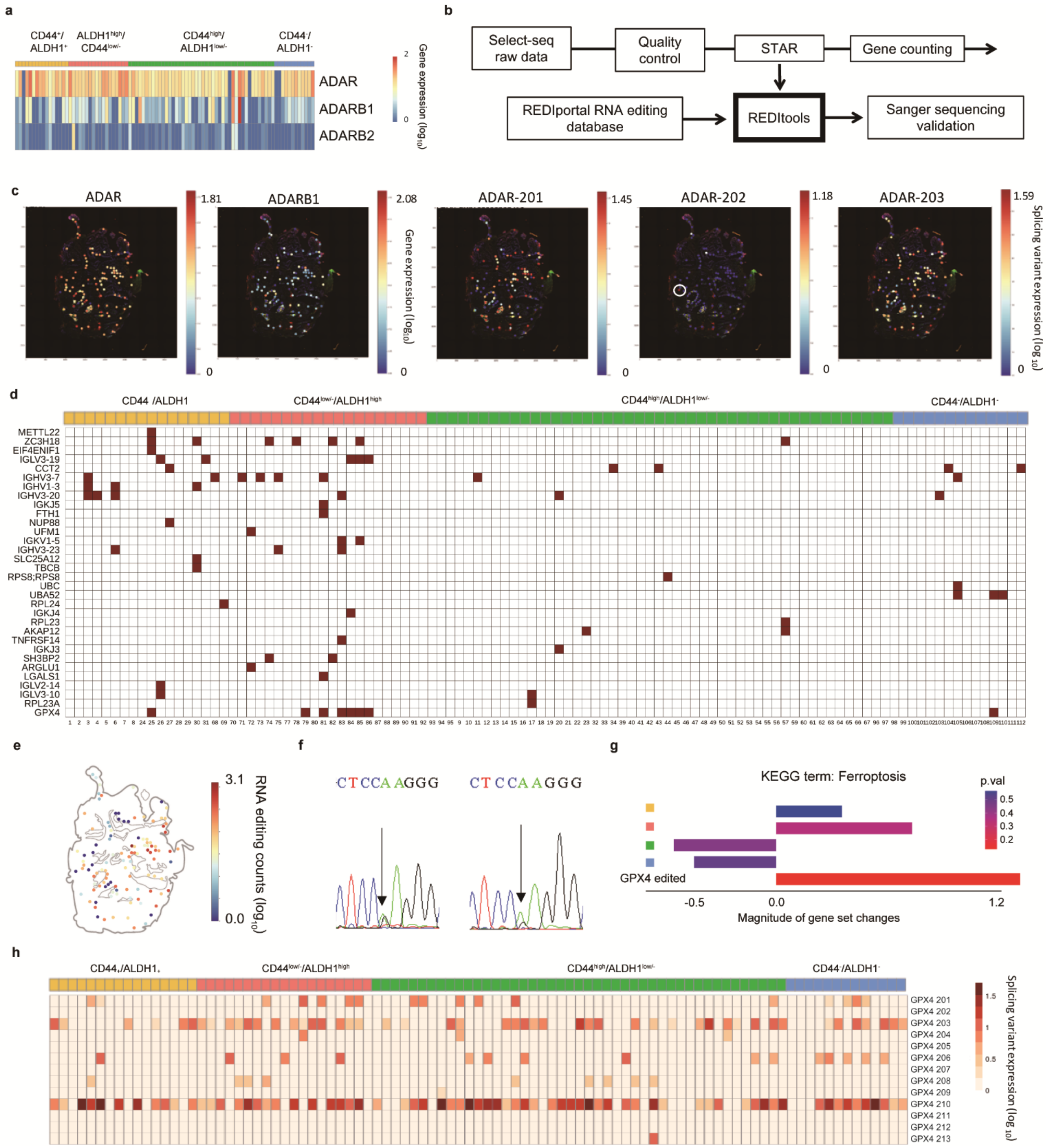

Supplementary Fig. 5. ADAR gene or transcript expression heatmap of the target regions and A-to-I editing events induced by ADAR. a, Gene expression heatmap of *ADAR*, *ADARB1*, and *ADARB2*. Green, red and blue marks indicate CD44-positive and ALDH1-negative/low;

ALDH1-positive and CD44-negative/low; and CD44-low and ALDH1-low regions determined by IF. **b,** Workflow of deducing A-to-I editing events using REDItools. **c,** Spatial heatmap of expressed *ADAR* isoforms. The *ADAR* gene transcript variants mostly consisted of *ADAR*-201 and *ADAR*-203, but in some of the $CD44^{low/-}$/$ALDH1^{high}$ regions that had high *ADAR*-201 and *ADAR*-203 expression, other transcript variants of *ADAR*, such as 204, 208, and 209, were also expressed. **d,** Heatmap of A-to-I editing events in exonic regions. **e,** Spatial mapping of the total number of A-to-I events in tissue A. **f,** Sanger sequencing validation examples. **g,** Gene set enrichment analysis (GSEA) between four staining groups and A-to-I edited samples using the Kyoto Encyclopedia of Genes and Genomes (KEGG) term ferroptosis. **h,** Expression heatmap of the alternative splicing variants of the *GPX4* gene.

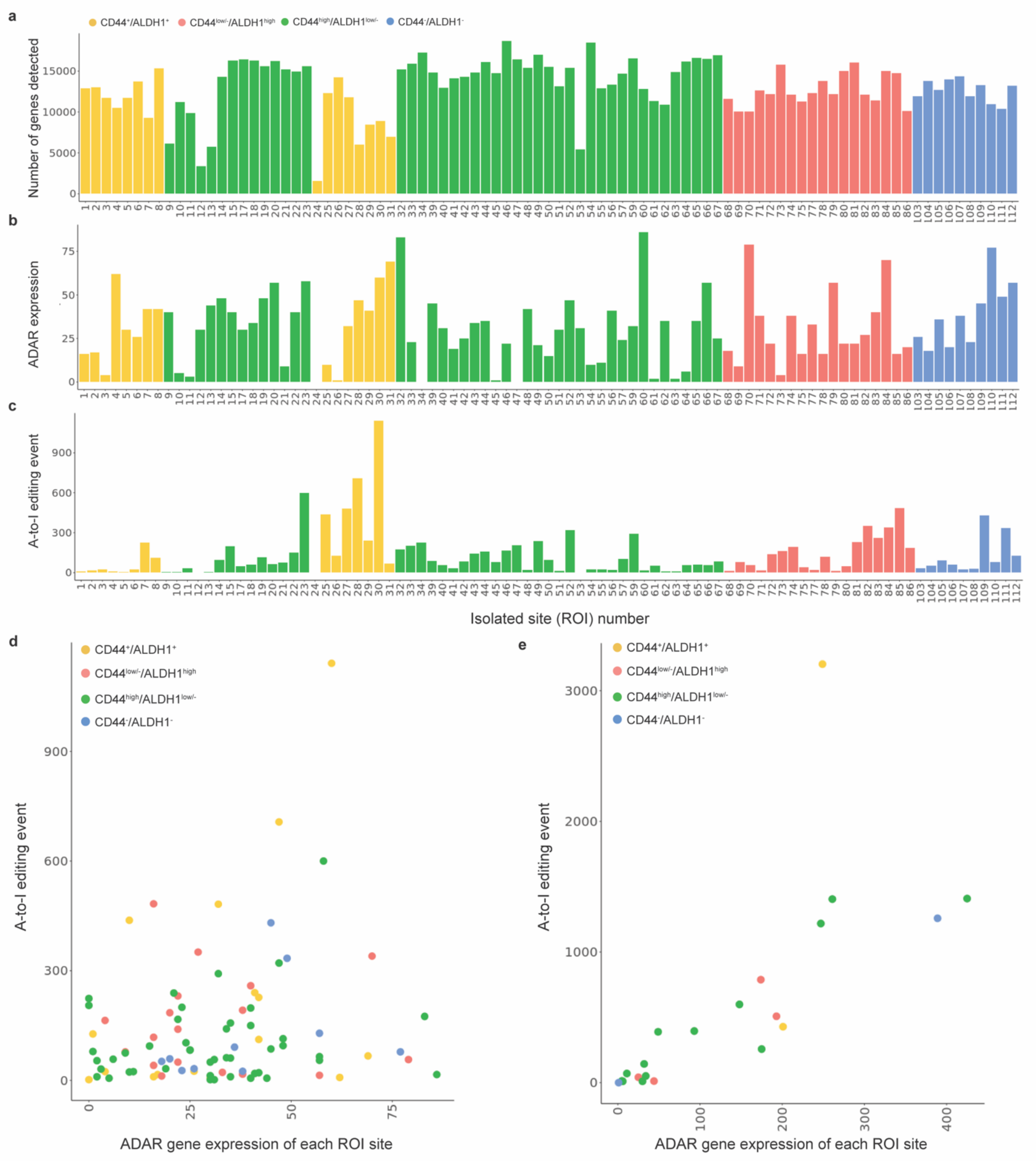

Supplementary Fig. 6. Correlation of ADAR expression with A-to-I editing for each ROI site. a, Number of genes detected for each ROI site. **b,** Number of A-to-I editing event for each ROI site. **c,** Correlation between the ADAR expression with A-to-I editing event for each ROI site. **d,** Correlation between the ADAR expression with A-to-I editing event for each IF group.

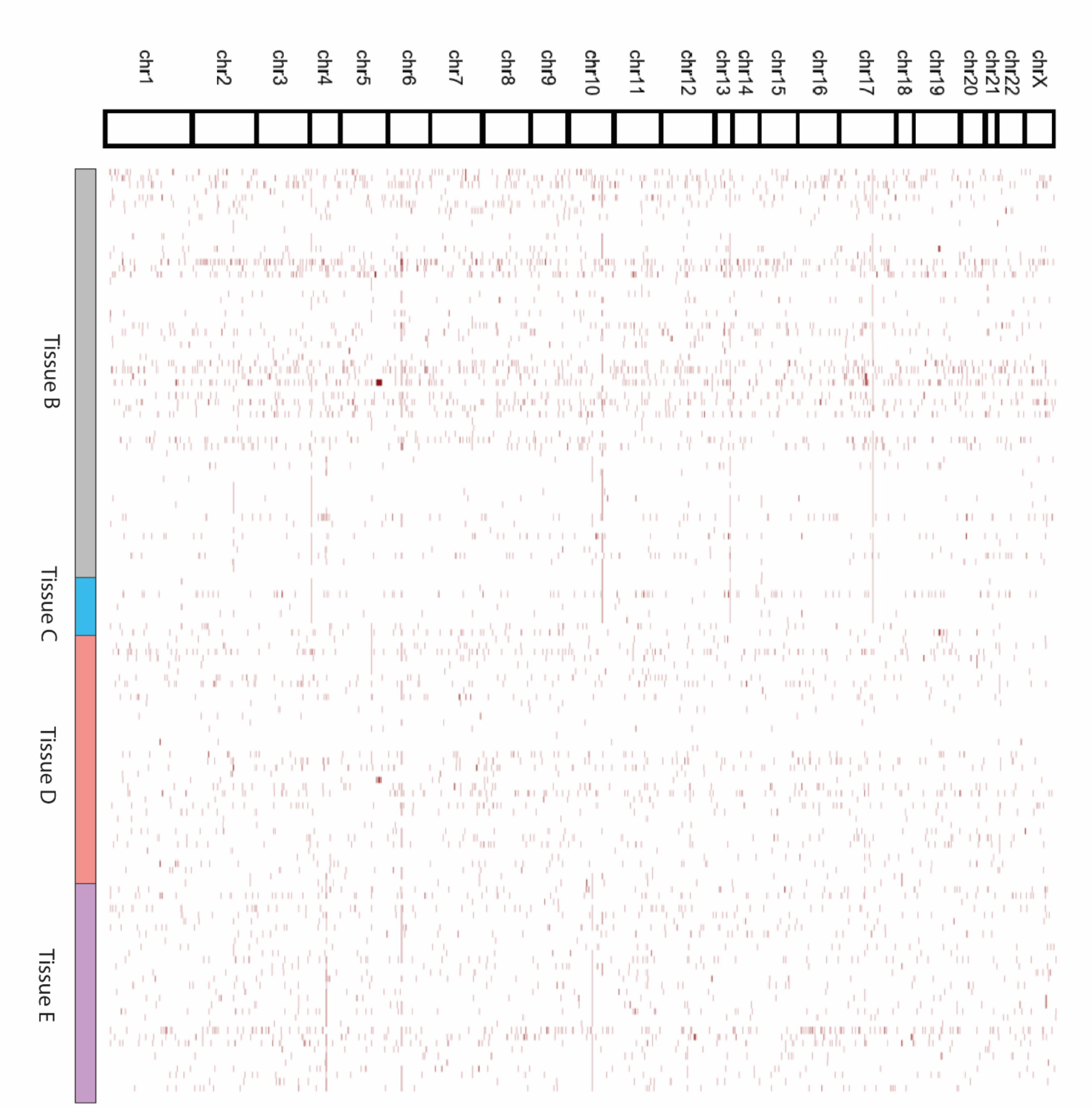

Supplementary Fig. 7. Spatial A-to-I editome landscape in four different tissues from four TNBC patients. Source data are provided as a Source Data file. A-to-I editome marks signature editing sites from different tissue and different group of microniches within the same tumour.

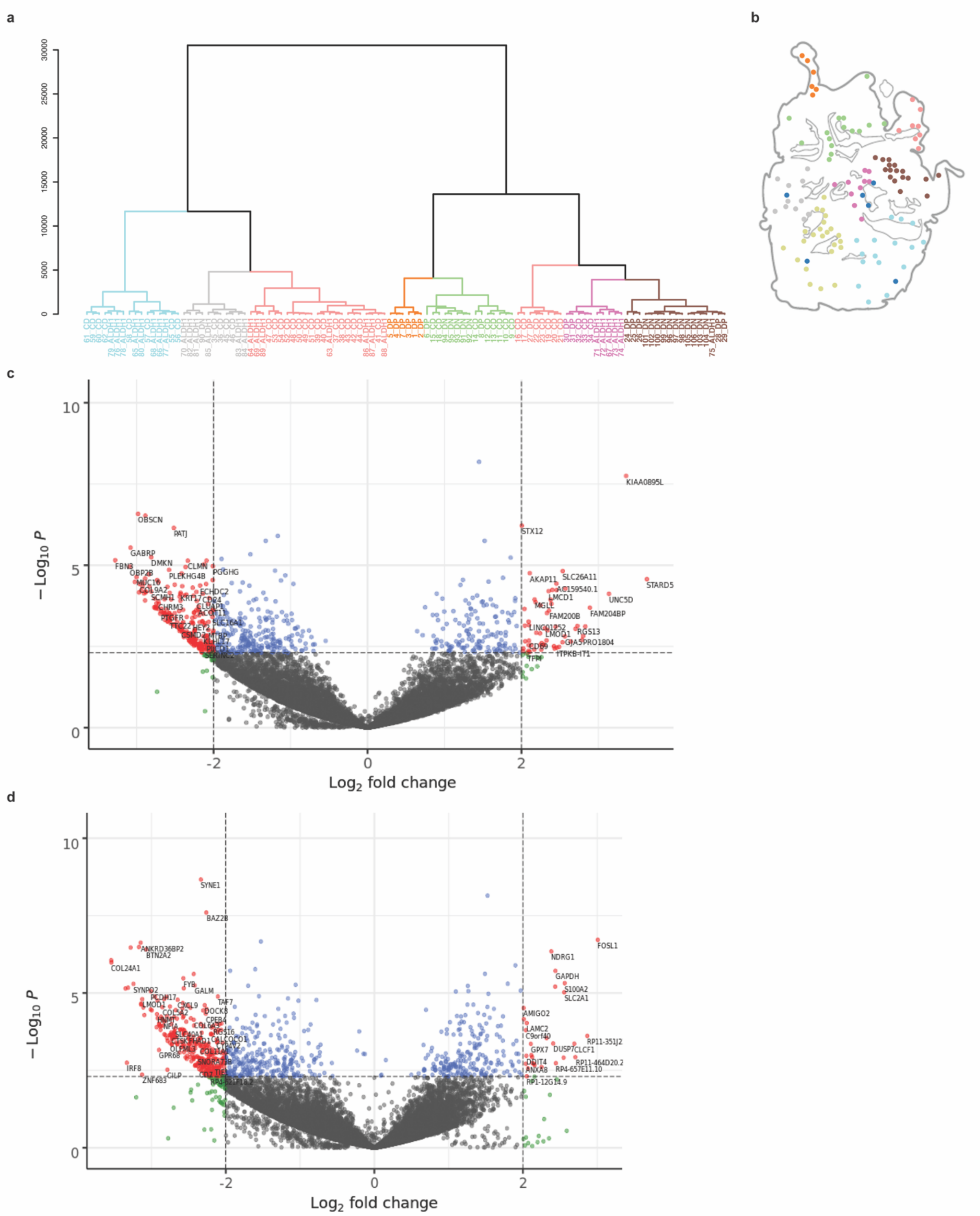

Supplementary Fig. 8. Spatial clustering result of each ROI site and differential expression analysis of spatial cluster groups. a, Hierarchy tree of agglomerative clustering results using

both spatial position and gene expression of each ROI site. **b,** Embedded spatial cluster group on tissue A. **c,** Differential expression analysis results of spatial cluster group 1 versus the other spatial cluster groups. **d,** Differential expression analysis results of spatial cluster group 3 versus the other spatial cluster groups.

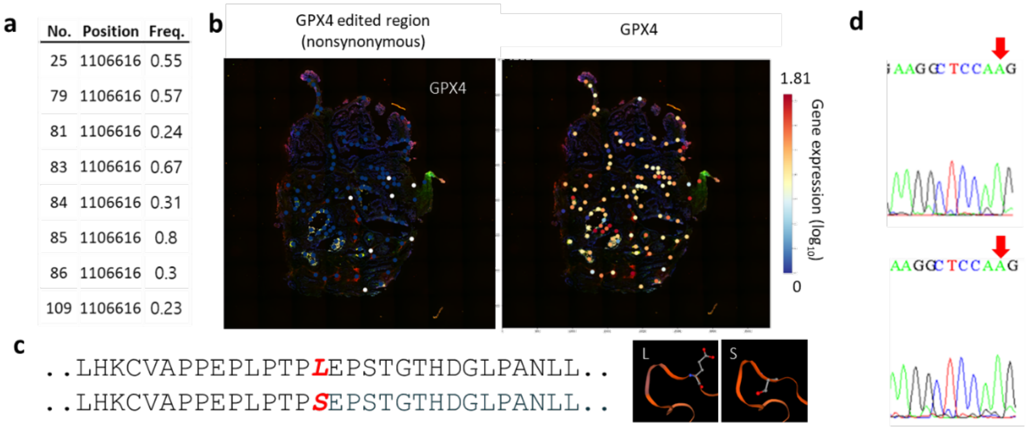

Supplementary Fig 9. GPX4 A-to-I editing characterization **a,** Different GPX4 expression frequencies. **b,** Distribution of GPX4 edited regions and GPX4 expression show little correlation. **c,** A-to-I edited amino acid residue and altered protein structure simulation. **d,** Genomic DNA validation shows only adenosine sequence at the site 1106616 of GPX4 gene.

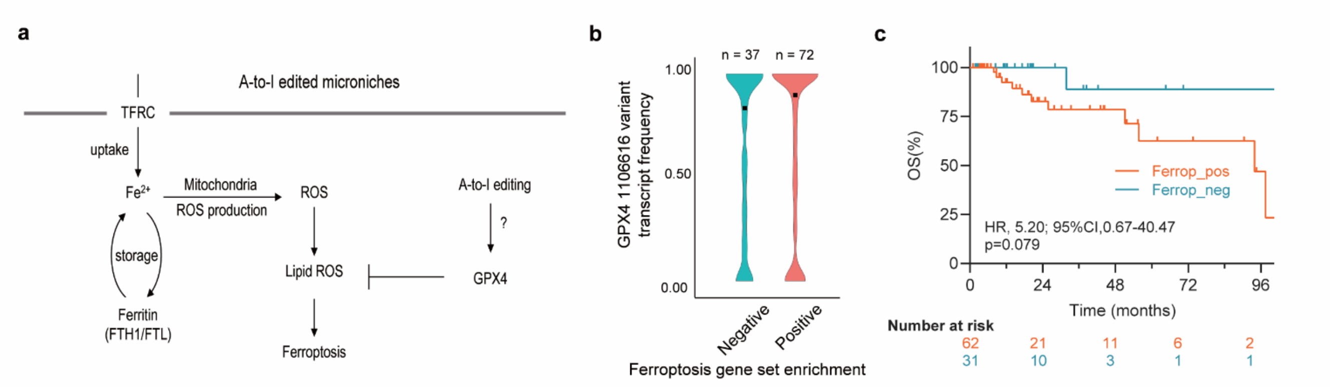

Supplementary Fig. 10. A model for how A-to-I editing affects GPX4 gene expression and ferroptosis and assessment of TCGA data set. Source data are provided as a Source Data file. **a,** A model of how A-to-I editing of *GPX4* affects ferroptosis. **b,** *GPX4* 1106616 variant comparison among patients with bulk transcriptome data in The Cancer Genome Atlas (TCGA) (n=109). The bulk transcriptomes with negative ferroptosis gene set enrichment values are grouped in blue, and those with positive values are grouped in red. **c,** Kaplan-Meier survival analysis of patients grouped the same as in b. P-values were calculated through two-sided log-rank test.

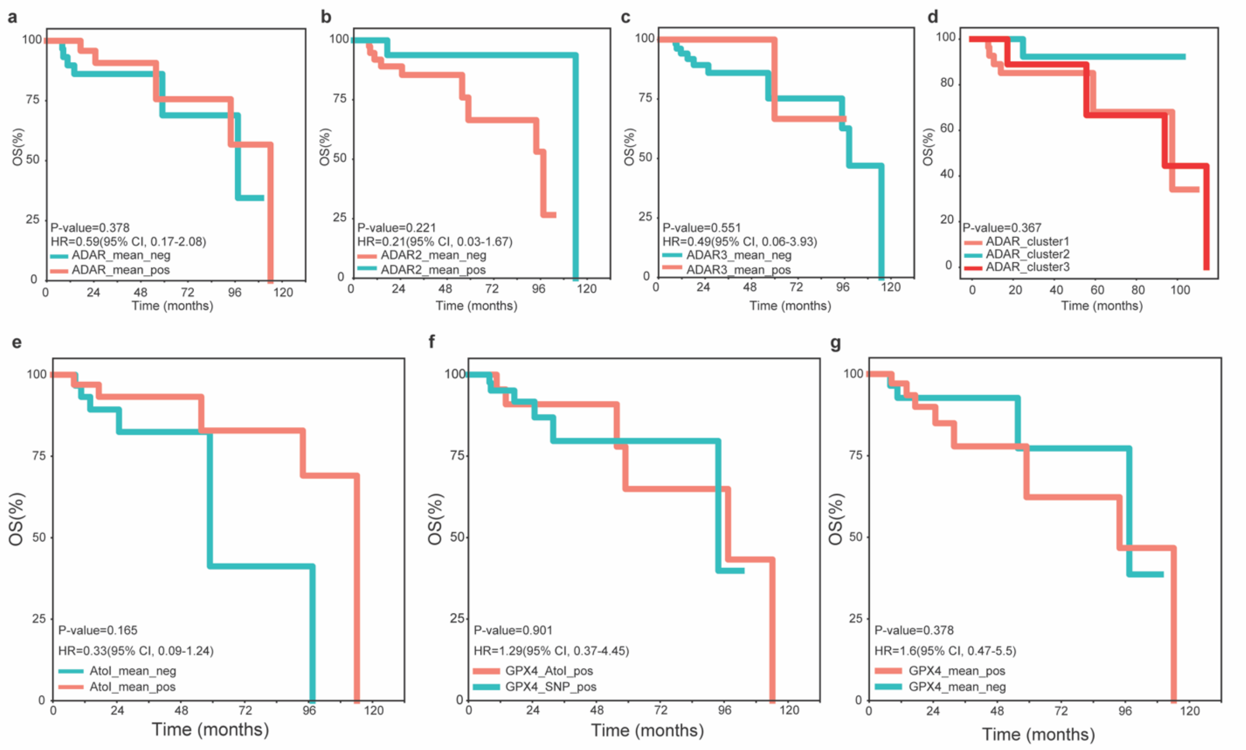

Supplementary Fig. 11. Correlation between survival of TNBC patients with various conditions. All P-values were calculated through two-sided log-rank test. **a,** Correlation of ADAR1 expression with survival. TNBC patients were group based on the mean ADAR1 expression of total patients. Source data are provided as a Source Data file. **b,** Correlation of ADAR2 expression with survival. TNBC patients were group based on the mean ADAR2 expression of total patients. **c,** Correlation of ADAR3 expression with survival. TNBC patients were group based on the mean ADAR3 expression of total patients. **d,** Correlation of combination of ADAR expression with survival. ADAR groups were clustered by agglomerative clustering using ADAR expression. **e,** Correlation with A-to-I editing event with survival. TNBC patients were group based on the mean number of A-to-I event. **f,** Correlation with GPX4 with A-to-I editing and SNP. TNBC patients were group based on the exist of A-to-I event and SNP on GPX4. SNP status were identified based on the normal blood exome sequencing data of TCGA. **g,** Correlation with GPX4 A-to-I editing frequency.

Supplementary Table 1. Comparison of Select-seq to existing technologies.

**Full-length transcriptome methods**	**LCM-seq Nichterwitz, et al. Nature communications (2016)**	**Geo-seq Chen et al. Nature protocols (2017)**	**Advanced LCM-seq Baccin et al. Nature cell biology (2020)**	**Select-seq (this work)**
**Speed (Throughput)**	3 targets in more than 1 HOUR[1]	3 targets in more than 1 HOUR[1]	3 targets in more than 1 HOUR[1]	6,000 targets Within 1 HOUR
**Size (Number of cells isolated from tissue)**	100-200 cells (130 ~750 μm^2)	up to 20 cells (~1,000 μm^2)	200-300 cells (14,500 μm^2)	5-10 cells (10~100 μm^2)
**Quality of data after staining (Compatibility to conventional pathology)**	X (Ethanol fixation and Nucleic acid (Nissl) Antibody (IHC))	X (Ethanol fixation and Nucleic acid (Nissl))	O (Paraformaldehyde fixation and immunofluorescence staining)	O (Paraformaldehyde fixation and immunofluorescence staining)
**Quality of data (Sequencing reads per cell)**	~ 10,000 (total 1 million reads)	~ 25,000 (total 0.5 million reads)	~ 5,000 (total 1 million reads)	200,000 (total 1 million reads)

Supplementary Table 2. Spatial group list.

Spatial group number	Location	Immunofluorescence information
1	1, 2, 3, 4, 5, 6, 7, 8,	CD44^{+}/ALDH1^{+}
2	24, 25, 26, 27, 28, 29, 30, 31	CD44^{+}/ALDH1^{+}
3	68	CD44$^{low/-}$/ALDH1^{high}
4	69, 70, 71, 74, 83, 86, 95	CD44$^{low/-}$/ALDH1^{high}
5	75	CD44$^{low/-}$/ALDH1^{high}
6	72, 73, 77, 78, 79, 80	CD44$^{low/-}$/ALDH1^{high}
7	81, 82, 84, 85	CD44$^{low/-}$/ALDH1^{high}
8	14, 15, 16, 17, 18, 19, 20, 21, 22, 23	CD44^{high}/ALDH1$^{low/-}$
9	9, 10, 11, 12,13	CD44^{high}/ALDH1$^{low/-}$
10	32, 33, 34	CD44^{high}/ALDH1$^{low/-}$
11	41, 42, 43, 44, 45, 46, 47, 48, 49, 50	CD44^{high}/ALDH1$^{low/-}$
12	51	CD44^{high}/ALDH1$^{low/-}$
13	39,40	CD44^{high}/ALDH1$^{low/-}$
14	52, 53, 54, 55, 56	CD44^{high}/ALDH1$^{low/-}$
15	57, 58, 59	CD44^{high}/ALDH1$^{low/-}$
16	60, 61	CD44^{high}/ALDH1$^{low/-}$
17	62	CD44^{high}/ALDH1$^{low/-}$
18	63	CD44^{high}/ALDH1$^{low/-}$
19	64, 65, 66, 67	CD44^{high}/ALDH1$^{low/-}$
20	97, 98, 99, 100, 101	CD44^{-}/ALDH1^{-}
21	102, 103, 104, 105, 106, 107, 108, 109, 110, 111, 112	CD44^{-}/ALDH1^{-}

Supplementary Table 3. Tools for RNA-seq, Gene Ontology, T/B cell receptor and A-to-I editing analysis.

Tool	Functionality	Version
Cutadapt	Adapter trimming	3.1
STAR	Sequence alignment to reference genome	2.7.3a
RSEM	Transcript/Gene count (RPKM count)	1.2.25
featureCounts	Transcript raw read count	2.0.0
RSeQC	Gene coverage calculation	2.6.4
Qualimap	Exon region percentage calculation	2.2.1
DESeq2	Differential gene expression analysis	1.26
REDItools	A to I editing site calling	2
TraCeR/BraCeR	T cell receptor/B cell receptor calling from RNA-seq	0.6.0/x

Supplementary Table 4. Primers used for this study.

Oligo-dT primer	5’- AAGCAGTGGTATCAACGCAGAGTACT_{30}VN-3’
Template Switching Oligo (TSO)	5′- AAGCAGTGGTATCAACGCAGAGTACrGrG +G-3
PCR primer	5′-AAGCAGTGGTATCAACGCAGAGT-3′
